# The Admixture Structure and Genetic Variation of the Archipelago of Cape Verde and Its Implications for Admixture Mapping Studies

**DOI:** 10.1371/journal.pone.0051103

**Published:** 2012-11-30

**Authors:** Sandra Beleza, Joana Campos, Jailson Lopes, Isabel Inês Araújo, Ana Hoppfer Almada, António Correia e Silva, Esteban J. Parra, Jorge Rocha

**Affiliations:** 1 IPATIMUP, Instituto de Patologia e Imunologia Molecular da Universidade do Porto, Porto, Portugal; 2 CIBIO, Centro de Investigação em Biodiversidade e Recursos Genéticos, Vairão, Portugal; 3 Universidade de Cabo Verde, Cidade da Praia, Cabo Verde; 4 Department of Anthropology, University of Toronto at Mississauga, Mississauga, Ontario, Canada; 5 Departamento de Biologia, Faculdade de Ciências da Universidade do Porto, Porto, Portugal; State University of New York College at Oneonta, United States of America

## Abstract

Recently admixed populations offer unique opportunities for studying human history and for elucidating the genetic basis of complex traits that differ in prevalence between human populations. Historical records, classical protein markers, and preliminary genetic data indicate that the Cape Verde islands in West Africa are highly admixed and primarily descended from European males and African females. However, little is known about the variation in admixture levels, admixture dynamics and genetic diversity across the islands, or about the potential of Cape Verde for admixture mapping studies. We have performed a detailed analysis of phenotypic and genetic variation in Cape Verde based on objective skin color measurements, socio-economic status (SES) evaluations and data for 50 autosomal, 34 X-chromosome, and 21 non-recombinant Y-chromosome (NRY) markers in 845 individuals from six islands of the archipelago. We find extensive genetic admixture between European and African ancestral populations (mean West African ancestry = 0.57, sd = 0.08), with individual African ancestry proportions varying considerably among the islands. African ancestry proportions calculated with X and Y-chromosome markers confirm that the pattern of admixture has been sex-biased. The high-resolution NRY-STRs reveal additional patterns of variation among the islands that are most consistent with differentiation after admixture. The differences in the autosomal admixture proportions are clearly evident in the skin color distribution across the islands (Pearson r = 0.54, *P*-value<2e–16). Despite this strong correlation, there are significant interactions between SES and skin color that are independent of the relationship between skin color and genetic ancestry. The observed distributions of admixture, genetic variation and skin color and the relationship of skin color with SES relate to historical and social events taking place during the settlement history of Cape Verde, and have implications for the design of association studies using this population.

## Introduction

Populations with peculiar genetic structures offer unique opportunities for studying human population history and for understanding the genetic basis of complex traits. In particular, recently admixed populations that trace their ancestry to multiple continents are especially well suited for identifying genes for traits and diseases that differ in prevalence between parental populations [1]. Given their history of recent admixture, African-American populations have been the focus of numerous population genetic and admixture mapping studies [2]–[13]. Individual African ancestry distributions of African-American population groups from across the United States were shown to be highly skewed toward higher values, with mean African contributions varying between 70–95% [3], [8], [9], [11]–[13]. Despite this skewed distribution and less admixture than the theoretical optimal of 50% from each parental population, African-Americans have been used successfully in mapping studies of complex genetic traits like white cell count [6], [10], body mass index [2], and diseases like prostate cancer [4] and renal disease [5]. However, given the cultural and genetic heterogeneity of admixed groups, it is essential that multiple admixed populations are studied to fully appreciate the relationship between the genetic, historical and environmental determinants of those traits.

**Figure 1:**
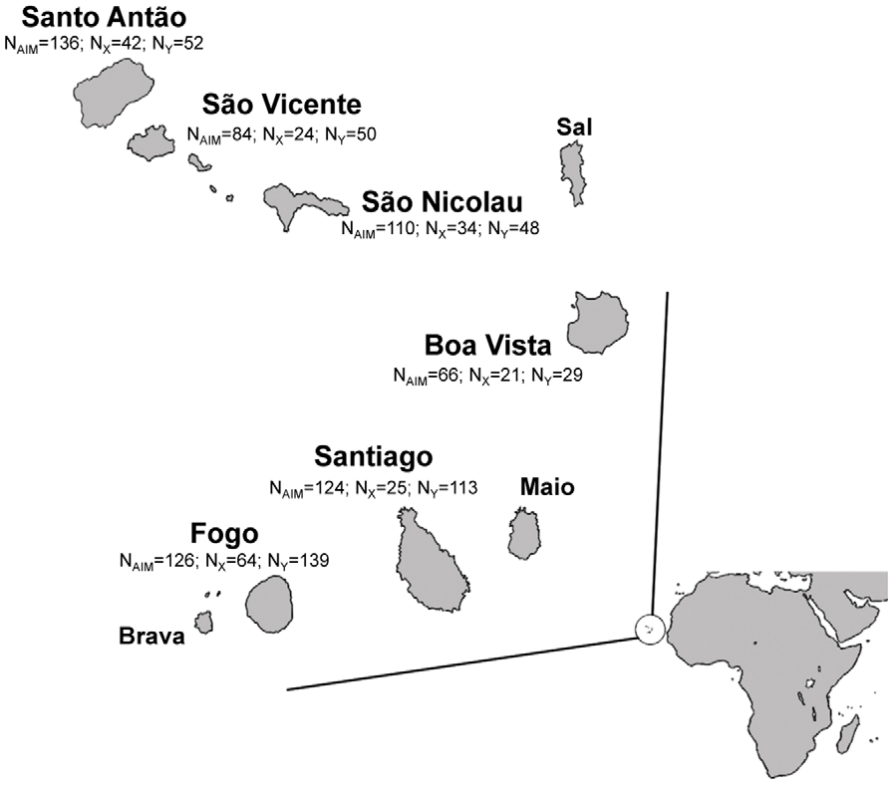
Map of Cape Verde. The number of individuals characterized for autosomal AIMs (N_AIM_), X-chromosome AIMs (N_X_) and NRY (N_Y_) is depicted for each sampled island.

The population of Cape Verde has great potential for admixture studies due to its well-documented history of contact between European colonizers and enslaved African peoples. Cape Verde is an archipelago located 450 km off the coast of Senegal, comprising ten islands that were uninhabited when first discovered by the Portuguese in the 1460s (Figure 1). The settlement process ensuing the initial discovery was mainly driven by the prospects of commercial trade with the Senegambian coast and may be conveniently divided into three major stages [14]. The first stage, encompassing the 15^th^ and the 16^th^ centuries, corresponds to the peopling of Santiago and Fogo islands, both located in the south of the archipelago (Figure 1). The original settlers (mostly Portuguese) occupied first the largest island of Santiago, which offered the best natural conditions to produce goods like cotton and horses that were exchanged on the African mainland for ivory, spices and slaves originating from regions extending from Senegal to Sierra Leone [15]–[17]. By 1480, landowners from Santiago had begun to settle in the nearby island of Fogo (Figure 1), to establish large cotton plantations and expand the trade with Africa. The majority of slaves, arriving in far greater numbers than the European colonizers, were exported to the Antilles, Central America, and Brazil [15], [18]. Slaves remaining in the islands were divided in two major groups: “rural slaves”, who were used to support the plantation system; and “domestic slaves”, mostly women, who were progressively integrated into the slave-owner households [15]. As a result of this assimilation process, the offspring of mixed unions between European men and African women soon became a predominant group within the non-enslaved segments of the early Cape Verdean society. The Cape Verde Creole language is the most significant cultural legacy of this admixture process and, according to historical sources, its use was likely to be generalized as early as the 1540s [16]. Unlike Santiago and Fogo, the other Cape Verde islands did not develop densely populated centers during the first settlement stage, and were mainly used for large-scale goat and cattle breeding, which did not require the continuous presence of a large labor force [14]. However, in the beginning of the 17^th^ century, due to increased competition with French and English slave traders, the slave-based economy of Santiago and Fogo had begun to decline and many free peasants were attracted by the good conditions for agriculture provided by the islands of Santo Antão and São Nicolau, in the North, and Brava, in the South (Figure 1). The steady occupation of these islands during the 17^th^ and 18^th^ centuries marked the beginning of the second settlement stage. In this stage, the absence of a significant slave labor force, the diversity of crops used in agriculture, and the small area of land detained by landowners strongly contrasted with the plantation system prevailing in Santiago and Fogo during the first peopling stage [14].

The third major colonization stage of Cape Verde corresponds to attempts to people the islands of São Vicente and Santa Luzia, in the northwest (Figure 1), under the direct stimulus of the Portuguese Crown, in the end of the 18^th^ century [14], [19]. However, these arid islands lacked water sources and suitable soils for agriculture, and all peopling efforts were doomed to failure. It was only in the mid 19^th^ century, with the advent of commercial Atlantic shipping and the opening of Mindelo’s harbor, that São Vicente was effectively peopled, rapidly becoming the second most important island of the archipelago, after Santiago, in terms of population size [20].

The eastern islands of Sal, Boa Vista, and Maio (Figure 1), remained somewhat disconnected from the major peopling events. Scarcely populated during the first stage, and without the good agricultural conditions of the islands peopled during the second stage, these three islands remained dedicated to cattle breeding and salt harvesting until the opening of the Boa Vista and Maio harbors to English and North-American ships during the 18^th^ century [14]. However, these islands never became important demographic centers and the joint population size of Sal, Boa Vista and Maio represents only about 8.5% of the total population of the archipelago (Cape Verde National Institute of Statistics, www.ine.cv).

Previous work on the genetic composition of the Cape Verde islands, based on classic protein markers and autosomal Short Tandem Repeat (STR) loci, detected substantial levels of African-European admixture, with mean proportions of European ancestry ranging from 36 to 54%, depending on the markers and statistical methods used to quantify admixture [21], [22]. However, these studies did not provide individual ancestry estimates and paid little attention to variation in the amount of admixture across islands. Studies based on the uniparentally inherited lineages from the non-recombinant Y-chromosome (NRY) and mitochondrial DNA (mtDNA) confirmed the predominance of mixed unions involving European males and African females [23], [24]. These surveys also provided evidence that Cape Verde is not genetically homogeneous [23], [24], but they used predefined geographic groups that lumped together islands with different settlement histories [14], and, therefore, could not offer a full portrait of the patterning of genetic diversity in the Cape Verdean territory.

Despite the potential usefulness of Cape Verde for conducting admixture mapping studies, with the exception of a recent study on iris texture traits [25], there is no data on the extent of phenotypic variation in anthropologically and biomedically relevant characteristics within the archipelago. In particular, the lack of data on the relationship between skin pigmentation and individual ancestry stands in sharp contrast with the information available from other admixed populations, where skin color variation has been characterized both as a mediator of social interactions and a model phenotype for admixture mapping studies [26], [8], [27], [28], [11].

Here, we provide a more detailed picture of the patterns of genetic and phenotypic variation in Cape Verde by using objective skin color measurements, and a panel of 50 autosomal, 34 X-chromosome and 21 NRY markers to analyze a total of 845 individuals from six islands that were peopled across different settlement stages. In particular, we explore the following poorly studied aspects of the admixed structure and settlement history of the archipelago: i) the distribution of group and individual ancestry proportions across islands; ii) the relationship between genetic ancestry and skin pigmentation; iii) the interplay between skin color, genetic ancestry and socio-economic status (SES); and iv) the impact of population history on the degree of genetic differentiation between islands.

## Materials and Methods

### Population Samples

The population sample comprised 845 individuals from the islands of Santiago, Fogo, Santo Antão, São Vicente, São Nicolau, and Boa Vista (Figure 1). With the exception of Boa Vista, the sample includes the most populated islands of the archipelago.

A total of 646 individuals were characterized for autosomal genetic ancestry. X-chromosome diversity was studied in a subset of 210 males, and NRY diversity was studied in a subset of 232 men augmented with an extra set of 199 male individuals.

Information about age, SES indicators, and individual and parent place of birth was collected via questionnaire. To avoid including close relatives in our analysis, we also recorded the individual and both parents’ full name and inquired for acknowledge relationships between donors at each sampling location. With these procedures, we were able to detect pairs of parent/offspring, full siblings, half-siblings and avuncular relatives, of which we removed one individual from the analysis.

To minimize missing data we grouped individuals according to their own place of birth (4.8% of individuals had no knowledge about one of the parent’s, mainly the father’s, place of birth). However, the results were not significantly different from analyses based on combined self and parent’s place of birth.

All population samples were collected with informed consent according to procedures approved by the IPATIMUP Human Subjects Committee and by the National Ethical Committee for Health Research of Cape Verde.

### Skin Pigmentation

Objective skin color measurements were taken with the handheld narrow-band reflectometer DSMII ColorMeter (Cortex Technology, Denmark). The DSMII ColoMeter is an updated version of the DermaSpectrometer (Cortex Technology, Denmark) used in previous studies [8], [11], which holds a new design of the optics to ensure minimal sensitivity to environmental light.

The melanin content was quantified by the melanin index (M index) provided by the instrument, which equals 100×log (1/% reflectance at 655 nm) [29].

For each subject, three consecutive measurements were taken on the unexposed upper inner side of each arm and the six measures were averaged to yield a mean M index value per individual.

### Ancestry Informative Markers

To estimate population and individual admixture proportions, we selected a panel of 50 autosomal and 34 X-chromosome Ancestry Informative Markers (AIMs), showing allele frequency differentials (δ) >0.5 between Europeans and West Africans (mean 0.748, range from 0.53 to 1.00) [11], [30]. Detailed information about these panels is provided in Tables S1 and S2. Autosomal AIMS were genotyped using allele-specific PCR with universal energy transfer-labeled primers [31] at Prevention Genetics (Marshfield, Wisconsin, USA) and X-chromosome AIMS were genotyped using Sequenom iPLEX technology at the Gulbenkian Institute genotyping service (Lisbon, Portugal).

### NRY Markers

Samples were genotyped for 10 NRY unique event polymorphisms (Y-UEPs M213, M91, YAP, SRY4064, M2, M35, M78, M81, 12f2, and M269) with a hierarchical approach based in the Y-Chromosome Consortium (YCC) phylogeny [32], using direct reading of the PCR product in acrylamide gels, restriction fragment length analysis, direct sequencing, or allele-specific PCR, according to previously described methods [33], [34]. Eleven NRY Short Tandem Repeats (STRs; DYS19, DYS389I, DYS389II, DYS385, DYS390, DYS391, DYS392, DYS393, DYS437, DYS438, and DYS439) were genotyped in the same individuals with the Promega Powerplex Y System (Promega Corporation, Madison, Wisconsin, USA). Y-chromosome haplogroups defined by binary markers were named according to the most recent YCC guidelines [32].

### Statistical Analyses

#### Genetic ancestry

Group and individual ancestry estimates based in autosomal and X-chromosome AIMS were calculated with the software ADMIXMAP v3.7 for Windows [35], [36]. The program requires multilocus genotypes of the admixed individuals and the allele frequencies from each parental population. We specified a model with no “dispersion” of allele frequencies, in which the allele frequencies in the unadmixed populations (European and West African) are assumed to be identical to the corresponding ancestry-specific allele frequencies in the admixed population.

Since individual West African ancestry distributions were approximately normal in all islands, we examined differences in the distributions between islands with standard one-way analysis of variance (ANOVA). These analyses were performed in the R statistical computing environment (http://www.r-project.org/).

Male-specific ancestral contributions to the Cape Verdean gene pool were evaluated with binary NRY markers, using the ADMIX 2.0 program [37], not taking into account molecular distances between haplogroups, and assuming four parental populations: Iberian Peninsula, West Africa, North Africa and Sephardic Jews. In performing this 4-way ancestry analysis, we used the increased resolution of NRY to narrow down the European contribution to the Iberian Peninsula and further discriminate the contributions of northern Africans and Sephardic Jews, two populations also reported to have migrated to the islands [15], [16], [24]. Details about comparative dataset assembled for the NRY admixture analysis are provided in Table S3.

#### Relationship of skin color, ancestry and SES

Total skin M index distribution, and per island and per sex distributions were examined for normality and log-transformed to achieve an approximate normal distribution. Differences between islands and between sexes were assessed using one-way ANOVA. The relationship between age and skin color was assessed by the parametric Pearson correlation test. All these statistical analyses were performed in the R statistical computing environment.

We measured SES using 3 variables: self-reported education, occupation and household amenities. Education was assessed with a five-level ordinal categorical variable corresponding to: i) up to 2 years in school; ii) 6^th^ grade; iii) uncompleted high school; iv) completed high school or uncompleted college; v) completed college, vocational or professional training. For occupation, we created three non-ordered categories: 1) “white collar” professions, combining technical/managerial/administrative activities; 2) “blue collar” occupations, combining craftsman/machine operator/laborer and farming/fishing work; and 3) “other occupations”, including housewife and other occupations. Regarding household amenities, we performed a principal component analysis (PCA) on number-coded answers to 10 questions made in accordance to the census prepared by the Cape Verde National Institute of Statistics (www.ine.cv; see supplementary note for more details on the household amenities measured). Here we use scores on the two first principal components, which explain 64% of the variation in household amenity features.

To evaluate the relationship between skin pigmentation and genetic ancestry, we performed linear regression analysis on the estimated proportion of West African ancestry for the overall sample, including sex and age as covariates. The relationship among SES, skin color, and genetic ancestry, was evaluated through multiple regression analyses on the M index and on the estimated proportion of West African ancestry for the overall sample and for each island, including sex and age as covariates. The analyses were performed in the R statistical computing environment.

#### Genetic differentiation between the islands

We used the increased resolution of NRY in discriminating between closely related populations to assess the patterns of genetic differentiation within the archipelago. Pairwise F_ST_ and R_ST_ genetic distances for NRY binary marker haplogroups and STR-based haplotype variation, respectively, were calculated with the ARLEQUIN 3.11 software package [38]. Multidimensional scaling (MDS) based on F_ST_ and R_ST_ distance matrices was performed using the STATISTICA software package. Haplotype matching analyses among the islands were performed with the full STR set, excepting the DYS385 locus, due to its duplicated status. The relationships between NRY-STR based haplotypes sampled in different islands were assessed in networks constructed with NETWORK 4.5 software (http://www.fluxus-engineering.com). To resolve extensive reticulation, the reduced-median [39] and median-joining [40] algorithms were applied sequentially and intrahaplogroup variance-based weighting was used as previously described [41]. Chromosomes carrying NRY-STR allele duplications were omitted from the analysis.

## Results

### Variation in Population Admixture and Individual Ancestry Across Islands

#### Autosomal AIMS

**Figure 2:**
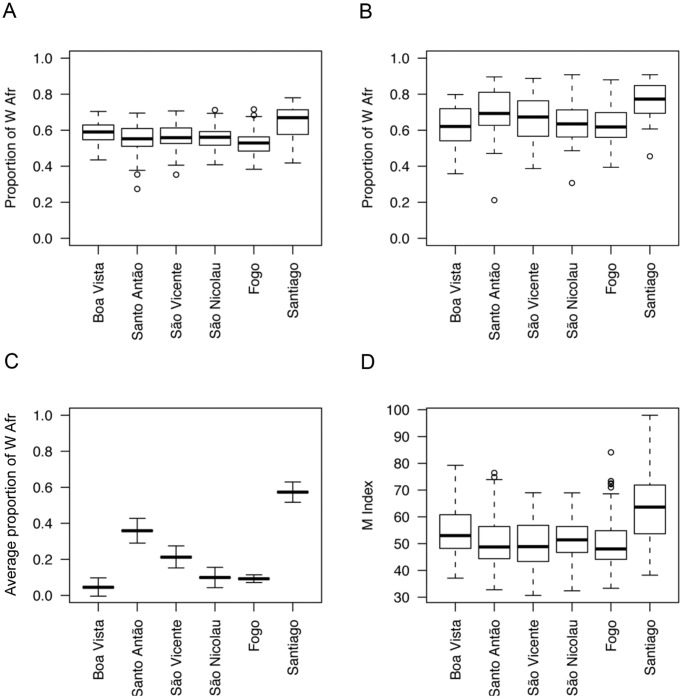
Admixture structure and skin color variation in Cape Verde. A-B) Individual West African ancestry distributions obtained with autosomal AIMs (A) and X-chromosome AIMS (B). C) Average group ancestry obtained with NRY-UEP markers. D) Skin pigmentation distributions among the Cape Verde islands.

The distribution of individual West African (WAfr) ancestry estimated with 50 autosomal AIMs in the six sampled islands from the Cape Verde archipelago is displayed in Figure 2A. The average proportion of West African admixture in the total sample of 646 individuals (0.57±0.08) is notably smaller than the average levels of 79–96.5% previously reported for African-American populations with a similar panel of markers [7], [8], [11] The data also clearly show that admixture levels are not uniformly distributed across the archipelago (*F* = 39.59, *df* = 6, *P* = 2.2e–16). The highest and lowest mean West African ancestry levels are found in Santiago (mean WAfr = 0.65; n = 124) and Fogo islands (mean WAfr = 0.53; n = 126), respectively (highest *P*-value found in one-way ANOVA comparisons with other islands is less than 0.0004). In the North (Figure 1), the islands of Santo Antão (n = 136), São Vicente (n = 84), and São Nicolau (n = 110), all with mean WAfr = 0.56, form a cluster with significantly lower West African ancestry than Santiago and Boa Vista (highest *P*-value 0.0006). Finally Boa Vista (mean WAfr = 0.59, n = 66) has an intermediate position, showing significantly higher individual West African ancestry values than the northern cluster and Fogo (highest *P*-value 0.0002).

#### X-chromosome AIMS

Figure 2B displays the distribution of individual West African (WAfr) ancestry calculated with 34 X-chromosome AIMs in 210 males from the six sampled islands. Consistent with the autosomal data, the mean level of African ancestry is highest in Santiago (mean WAfr = 0.76; n = 25). The other islands are clearly more admixed, with mean West African ancestry proportions ranging from 0.62 in Boa Vista (n = 21) to 0.70 in Santo Antão (n = 42), and showing intermediate levels in Fogo (0.63; n = 64), São Vicente (0.67; n = 24) and São Nicolau (0.64; n = 34). Overall, the proportions of African ancestry calculated with the X-chromosome are significantly higher than those obtained with the autosomal markers (Figure 2A and B; Wilcoxon signed rank test *P*-value* = *1.8e–05*),* confirming that the admixture pattern was sex biased, mostly involving European man and African women.

#### NRY

**Figure 3:**
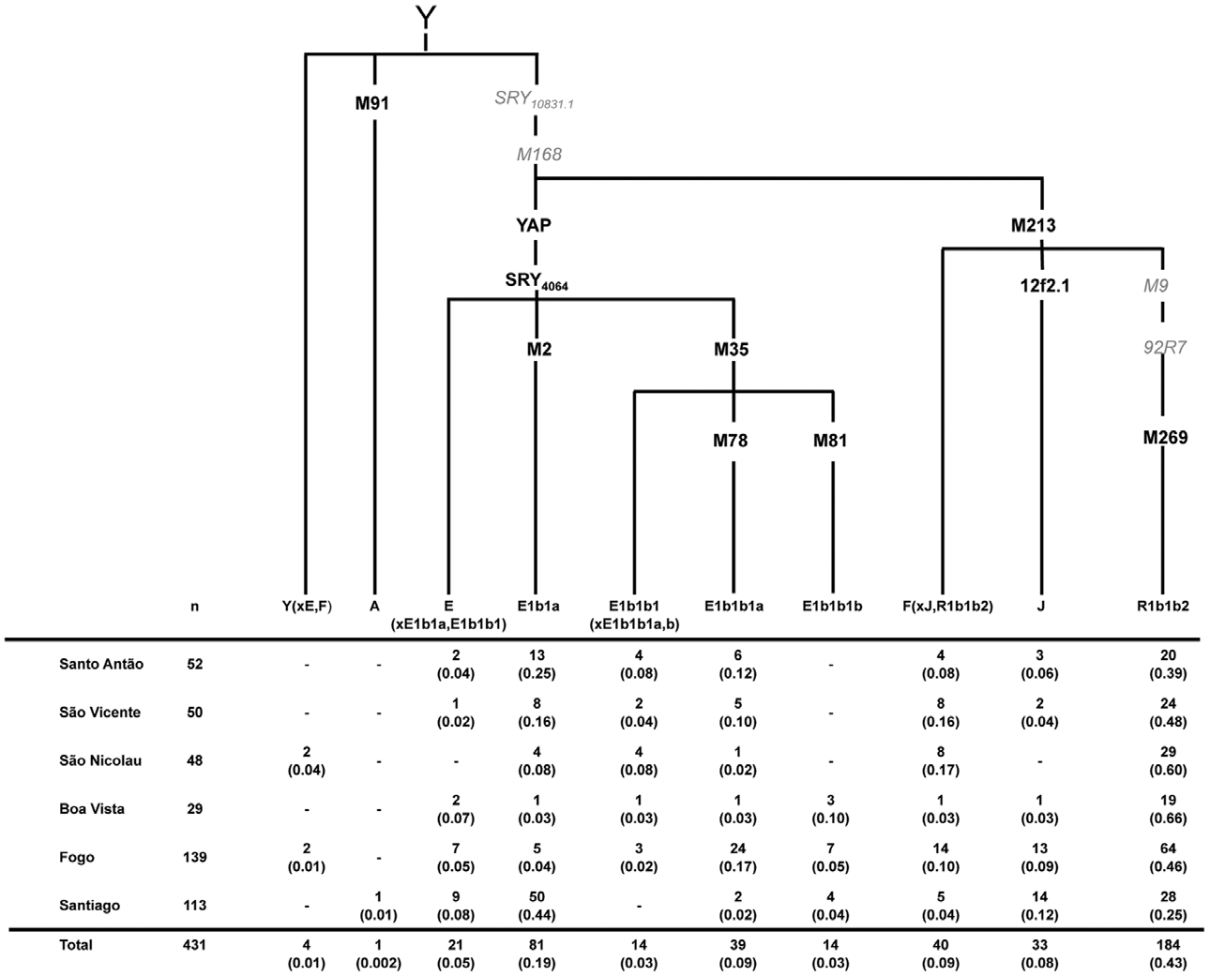
Phylogenetic tree of Y-UEP haplogroups studied in the Cape Verde sample. Haplogroup nomenclature as proposed by the YCC [32] and defining UEPs assayed are shown along the branches in *bold* and *black*. Mutations in *italics* and *grey* were not assayed in this studied. The table shows absolute frequencies (percentage) of the haplogroups found in each island and in the total archipelago.

Figure 3 shows the distribution of 10 NRY haplogroups defined by 10 binary markers in 431 males. The most frequent haplogroup in the total sample is R1b1b2 (42.7%), followed by E1b1a (18.8%); for all other haplogroups, frequencies are lower than 10%. R1b1b2 is the most common lineage in European populations, with frequencies ranging from 20% to 80% at the continental level [42] and from 59% to 66% in the Iberia Peninsula [43], [34]. E1b1a is typical of Africa, comprising ∼60–85% of NRY lineages in sub-Saharan populations, and specifically 81–85% in West African populations [33], [44]–[47]. The observed haplogroup distribution pattern confirms that the Cape Verdean paternal component is mainly derived from Europe, as previously reported [24].

To formally assess the paternal contribution of different populations, we performed an admixture analysis, using the approach implemented in Admix 2.0 [37] and treating the Cape Verdean population as a result of admixture of four parental populations: Iberian Peninsula, West Africa, North Africa and the Sephardic Jewish population (Table 1). This 4-way admixture analysis was prompted by previous suggestions, based on genetic and historical data, that enslaved North Africans and Iberian Jews represented non-negligible fractions of African and European parental groups, respectively [15], [16], [24].

**Table 1:** Estimated admixture proportions (± standard deviation) of Y-chromosome lineages from Cape Verde. Empty cells correspond to unsupported parental populations.

	Parental Population			
Hybrid Population	Iberian Peninsula	West Africa	North Africa	Sephardic Jews
Santo Antão	0.601±0.110	0.359±0.069	–	0.041±0.090
São Vicente	0.751±0.118	0.212±0.061	–	0.037±0.099
São Nicolau	0.901±0.056	0.099±0.056	–	–
Boa Vista	0.933±0.124	0.045±0.051	0.022±0.111	–
Fogo	0.743±0.073	0.092±0.022	0.104±0.041	0.060±0.065
Santiago	0.342±0.070	0.573±0.056	0.0004±0.035	0.084±0.061
Total	0.680±0.043	0.266±0.023	0.028±0.021	0.026±0.035

According to the admixture analysis, the majority of male contributions to Cape Verde were derived from the Iberian Peninsula (0.68). The second most important contribution (0.27) came from West Africa, while contributions from Northern Africa and Sephardic Jews seem to have been residual (∼0.03 each, with wide confidence intervals).

As with the autosomal and X-chromosome data, NRY-based admixture estimates are not homogeneous across islands (Table 1; Figure 2C). Santiago is again the island with the highest mean level of West African ancestry (0.57), while Fogo, in spite of its proximity to Santiago, has a much lower African contribution (0.09) that is closer to Boa Vista (0.05) in the East, and to São Nicolau (0.1) in the North (Figure 1). Santo Antão (0.36) and São Vicente (0.21), also in the North, have larger NRY African levels that are intermediate between those of Santiago and of the other islands.

### Relationship of Individual Ancestry and Skin Pigmentation

The overall distribution of skin pigmentation as measured by the melanin (M) index, ranges from 29.6 to 97.9 with a mean of 53.4 (median of 51.3). Because we measured skin color with an updated version of the reflectometer employed in previous studies of African-Americans populations [8], [11], our data are not directly comparable to these studies.

Skin pigmentation levels do not differ between sexes (male average skin M index = 53.8; female average skin M index = 53.2; *P* = 0.622), and are not correlated with age (*P* = 0.813), but are clearly different among islands (*F* = 21.603, *df* = 6, *P*-value = 2.2e–16), mimicking the patterns of variation in autosomal West African ancestry levels (Figure 2A and 2D). Individuals from Santiago are significantly darker than individuals from the other islands (mean skin M index = 63.2; highest *P*-value 2.21e–5 in one-way ANOVA pairwise comparisons). The neighboring island of Fogo (mean skin M index = 50.2) harbors the lightest skin M values, together with Santo Antão (mean skin M index = 50.6), São Vicente (mean skin M index = 50.2) and São Nicolau (mean skin M index = 51.6) in the North (Figure 1). Boa Vista island stands in an intermediate position (mean skin M index = 54.8), being significantly darker than Fogo and the northern islands (highest *P*-value 0.034), but significantly lighter than Santiago (*P*-value 2.2e–05).

In the total Cape Verde sample, skin pigmentation is significantly correlated with individual ancestry, with a clear trend towards darker pigmentations with increasing levels of West African ancestry (Pearson *r* = 0.54, *P*-value <2e–16; including sex as a covariate). Although our panel of autosomal AIMs includes five markers located within pigmentation candidate genes (Table S1), this correlation is still significant after removing these loci from the calculations, (Pearson *r* = 0.49, *P*<2e–16).

### Relationship between SES with Genetic Ancestry and Skin Pigmentation

To evaluate the relationships among SES, genetic ancestry, and skin pigmentation, we performed multiple regression analyses considering education (five ordered categories), occupation (three non-ordered categories) and household amenities (PC1 and PC2 from PC analysis of 10 categories) as dependent variables, and individual West African ancestry based on autosomal data and skin color as independent variables, while controlling for sex and age (Table 2). In the total sample all three SES measures were significantly associated with skin color, but not with the proportion of West African ancestry. There is an inverse relationship between skin color and education, occupation status, and the quality of household amenities.

**Table 2:** Multiple regression coefficients (β) and significance value (*P*) for regression of SES on skin color (M index), proportion of West African ancestry (Wafr), sex and age.

	North Cluster		Boa Vista		Fogo		Santiago		Total	
	β	*P*	β	*P*	β	*P*	β	*P*	β	*P*
**House.PC1** a										
Constant	−2.911	0.022	0.225	0.873	0.483	0.844	−2.119	0.018	−4.15	3.30e−06
Sex (male = 0, female = 1)	−0.068	0.525	−0.237	0.378	0.18	0.417	−0.186	0.410	−0.076	0.382
Age (years)	−0.001	0.820	0.015	0.255	0.004	0.72	0.012	0.266	0.002	0.584
M index	0.609	0.077	0.023	0.157	0.287	0.663	0.026	0.011	1.04^c^	5.60e−05
Wafr	0.678	0.427	−3.132	0.234	−3.019	0.101	0.719	0.655	−0.01	0.988
**House.PC2** a										
Constant	2.507	0.004	2.378	0.040	1.967	0.129	0.738	0.147	2.303	4.50e−05
Sex (male = 0, female = 1)	0.126	0.085	−0.250	0.250	0.092	0.431	−0.041	0.751	0.057	0.294
Age (years)	0.004	0.219	−0.006	0.562	−0.007	0.216	−0.01	0.093	−0.001	0.788
M index	−0.752	0.001	−0.016	0.222	−0.287	0.406	−0.014	0.016	−0.598^c^	0.0003
Wafr	0.364	0.531	−1.746	0.410	−1.139	0.238	0.726	0.430	0.107	0.788
**Occupation** b										
Constant	14.468	3.00e-−04	1.310	0.740	31.889	0.0006	6.256	0.002	17.895	4.30e−13
Sex (male = 0, female = 1)	−0.881	0.008	−0.007	0.992	−2.556	0.006	−0.434	0.357	−0.85	0.0002
Age (years)	−0.084	2.76e−08	−0.086	0.033	−0.165	0.0001	−0.057	0.014	−0.088	3. 8e−16
M index	−2.492	0.021	−0.047	0.245	−7.948	0.001	−0.089	0.0002	−3.74	5.70e−08
Wafr	−0.867	0.723	6.873	0.335	13.089	0.06	2.946	0.395	1.732	0.282
**Education**										
Constant	4.108	3.16e−07	1.290	0.138	4.024	0.002	2.246	6.20e−08	3.527	1.30e−12
Sex (male = 0, female = 1)	0.048	0.475	0.082	0.623	0.044	0.687	0.075	0.482	0.056	0.24
Age (years)	−0.016	8.86e−07	−0.018	0.018	−0.022	0.0004	−0.014	0.011	−0.017	6.90e−14
M index	−0.621	0.004	0.002	0.869	−0.629	0.07	−0.013	0.013	−0.519	0.0003
Wafr	−0.158	0.762	0.292	0.854	0.2	0.829	0.108	0.886	0.221	0.521

a PC1 and PC2 were estimated on the basis of 10 household amenity categories.

b White collar occupations are associated with smaller M-index values.

c Inspection of PC plots shows an inverse correlation between M index and quality of household amenities.

Because admixture proportions and skin color are not homogeneous across islands, we also tested for associations in each island (Table 2). For this analysis, we clustered the northern islands of Santo Antão, São Vicente, and São Nicolau in a single group, since admixture proportions and skin color distributions did not vary significantly among these islands. Except for Boa Vista, which has the lowest sample size (n = 66), skin color was associated with at least two indicators of SES in each island.

### Genetic Differentiation between Islands

**Figure 4:**
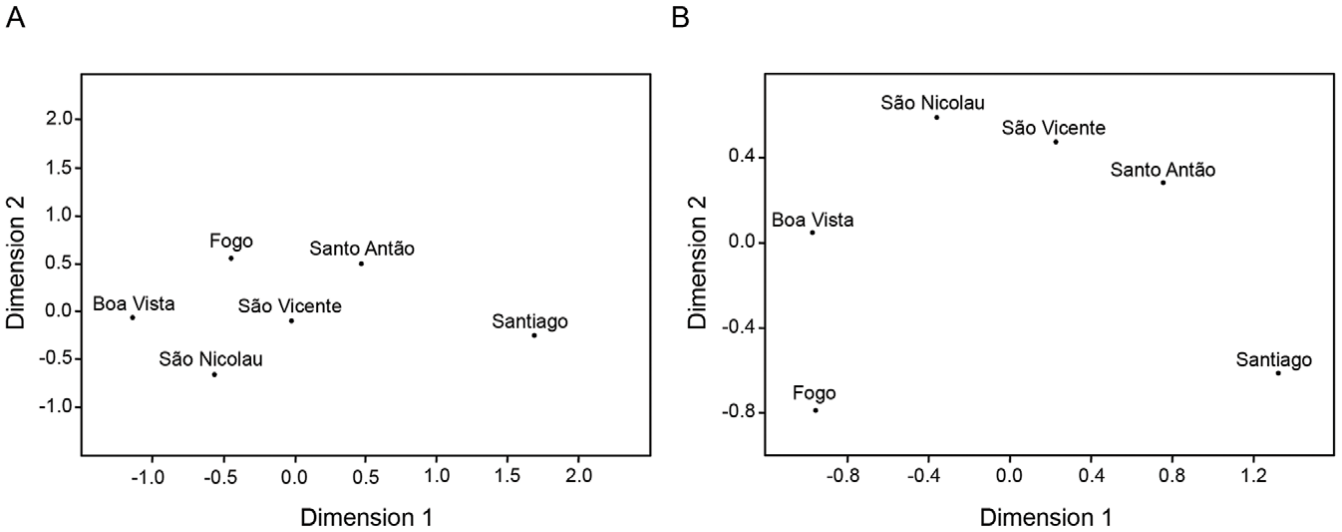
MDS plots illustrating the genetic structure within the Cape Verde archipelago. A) MDS plot of the F_ST_ genetic distance matrix estimated from Y-UEP haplogroup data (Stress value = 0.0000, *p*<0.01). B) MDS plot of the R_ST_ genetic distance matrix estimated from NRY-STR haplotype data (Stress value = 0.0000, *p*<0.01). Stress value significance was assessed as according to [60].

To further evaluate the relationships among different islands, we performed MDS analyses using pairwise genetic distances based on NRY haplogroups defined by binary markers, and NRY haplotypes defined by STRs (Figure 4). In the MDS plot calculated from F_ST_ distances and haplogroup frequency data, Santiago is clearly distinguished from the other islands in the first dimension, reflecting its higher levels of West African male ancestry (Figure 4A; Table 1). The plot based on R_ST_ distances and NRY haplotypes provides a better resolution of the differences among islands by uncovering additional genetic structure that is less related to the admixture process (Figure 4B). As with Y-UEP data (Figure 4A), the first axis of this plot separates Santiago from the other islands and most likely reflects differences in admixture proportions across the archipelago (Figure 4B). However, the second axis has a North-South geographic orientation, showing that islands with similar levels of admixture may harbor different NRY lineage profiles. Overall, the MDS plot for NRY haplotype variation is somewhat reminiscent of the geographic map of the archipelago, separating Fogo and Santiago from each other and from São Antão, São Vicente, and São Nicolau, which form a group of islands in the northern part of the archipelago (Figures 1 and 4B), with Boa Vista standing in an intermediate position.

To further trace the spread of STR-defined lineages across the archipelago, we have also studied the patterns of haplotype sharing among islands. The highest percentage of individual Y-chromosomes with no matches on other islands was found in Santiago (81%), followed by Boa Vista and São Vicente (51%), Fogo (38%), São Nicolau (35%), and Santo Antão (31%). Table 3 shows, for each pair of islands, the proportions of shared NRY lineages sampled in one island (rows) that are also observed in the other island (columns). For example, as much as 81% of shared individual Y-chromosomes from Fogo are also observed in Santiago, while only 62% of shared chromosomes sampled in Santiago were found in Fogo, showing that this island harbors a subset of Santiago’s NRY variation, despite the significant divergence in the genetic composition and admixture structure of the two islands (Figures 2 and 4). Moreover, most of Fogo’s lineage matches with Santiago are exclusive (Figure S1). This pattern likely reflects the first population movement within Cape Verde, involving the colonization of Fogo by Santiago inhabitants during the first peopling stage. A similar asymmetry in lineage sharing patterns suggests that the inhabitants of São Nicolau had an important role in the settlement of Boa Vista (Table 3). Santo Antão and São Vicente, the two closest inhabited islands in the archipelago (Figure 1), also have high levels of haplotype sharing (60–64%), consistent with the historically documented colonization of São Vicente with settlers from Santo Antão, followed by subsequent gene-flow between the two islands [20]. In general, the lineages sampled in the three northern islands and Boa Vista have lower levels of haplotype sharing with both Santiago and Fogo (Table 3), in accordance with the North-South discrimination observed in the second dimension of the MDS plot based on R_ST_ genetic distances (Figure 4B). However, lineages from São Vicente and São Nicolau still show moderate sharing levels with Fogo (∼43%), suggesting that this island might have been an important source of settlers in the northward migrations during the second colonization stage of the archipelago.

**Table 3:** Proportion of NRY-STR haplotypes sampled in one population (rows) that are also found in other populations (columns).

Island	Santo Antão	São Nicolau	São Vicente	Fogo	Santiago	Boa vista
Santo Antão	-	0.417	0.638	0.25	0.25	0.194
São Nicolau	0.464	–	0.393	0.428	0.25	0.357
São Vicente	0.608	0.304	–	0.434	0.217	0.13
Fogo	0.175	0.209	0.198	**–**	0.814	0.128
Santiago	0.238	0.286	0.19	0.619	–	0.048
Boa vista	0.462	0.769	0.308	0.231	0.077	–

**Figure 5:**
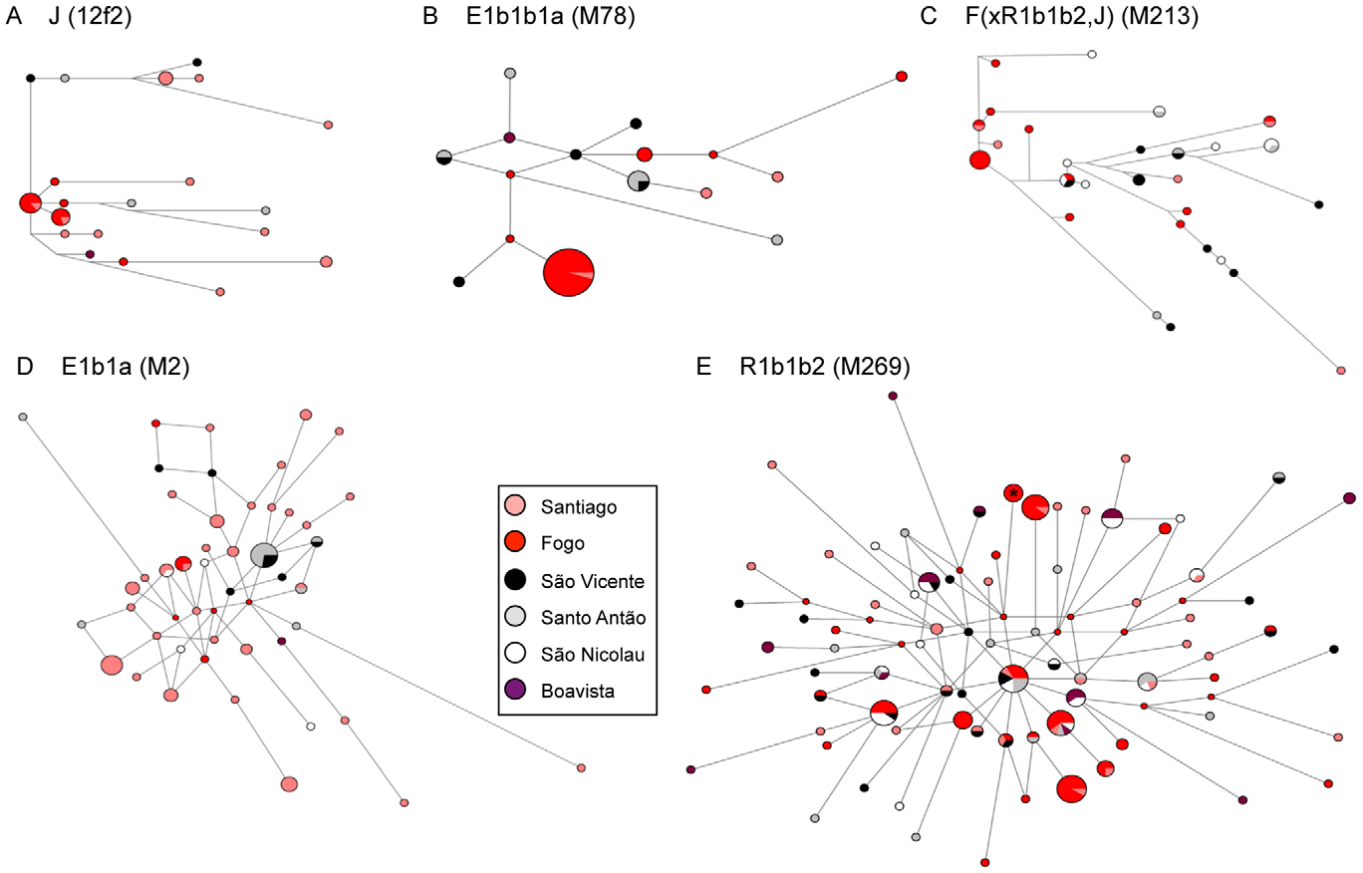
Median-joining (MJ) networks of the 11 NRY-STR haplotypes found within the most frequent Cape Verdean lineages. Circles represent haplotypes, with areas proportional to frequency; lines between circles represent NRY-STR mutational steps, with length proportional to haplotype mutational divergence. In E), the circle marked with an asterisk corresponds to the Montrond lineage in Fogo Island.

To better understand the phylogeographic relationships underlying lineage sharing patterns, we further compared the NRY-STR haplotype diversity within the most frequent haplogroups through network analysis (Figure 5). It is clear that Santiago harbors the more heterogeneous lineage composition, with a higher number of single and low frequent haplotypes than other islands. In contrast, the patterns of haplotype distribution within major lineages in Fogo [R1b1b2 (M269), J (12f2), F(xR1b1b2,J) (M213), and E1b1b1a (M78)] are strikingly the opposite and show clear signs of founding effects, with a relatively small number of different haplotypes, fewer rare haplotypes, and more haplotypes with intermediate frequencies (Figure 5). Intriguingly, one of Fogo’s most common lineages within the R1b1b2 haplogroup (4% in Fogo; marked with an asterisk in Figure 5) is associated with the surname “Montrond”, which was introduced in the island at the end of the 19^th^ century by the French immigrant Armand Montrond, who is known to have enjoyed a remarkably high reproductive success [48].

## Discussion

In this study we present an analysis of admixture and background population structure of the archipelago of Cape Verde using genetic information from autosomal and X-chromosome AIMS, and NRY-specific polymorphisms. The relevance of genotyped sample in terms of size and geographic coverage of the archipelago, as well as the large discriminating power of assayed markers allowed us to add substantial detail to the understanding of the historical factors that have shaped the patterns of genetic diversity within and among local island populations.

### Admixture Structure of the Archipelago of Cape Verde

Population-based admixture proportions estimated with 50 autosomal AIMs confirm our expectations based on historical evidence of extensive genetic admixture between Europeans and Africans in Cape Verde. As far as we know, Cape Verde is presently one of the most highly admixed populations resulting from the mixing of European and African parental contributions [7], [9], [11], [12], [49], [13], and may be only paralleled by some regions in Brazil [27], [50], [51]. Moreover, the comparison of African ancestry proportions calculated with X, Y-chromosome and autosomal markers confirms that admixture involved predominantly European men and African women, like in many other societies emerging from the Atlantic slave trade [33], [52], [27], [53].

The African ancestry proportions estimated with different panels of markers also revealed substantial variation in admixture among the sampled islands, with Santiago showing significantly higher levels of African ancestry than the other islands. This variation is generally consistent with the settlement history of the archipelago, since Santiago was the first island to be peopled and its economy was initially based on a plantation system that largely depended on African slaves [17]. In turn, the islands of Santo Antão, São Vicente, São Nicolau and Boavista, which show significantly lower African admixture levels than Santiago, were mostly populated by admixed free peasants that migrated northwards after the decline of the slave-based economy [17].

There is, however, an apparent discordance to this general pattern: Fogo island displays low African ancestry levels that are similar to the northern islands, even though its settlement history is concurrent with Santiago and based on the same slave labor system [17]. It is likely that this discrepancy resulted from differential survival and integration levels of “rural slave” communities after slavery was abolished. According to this interpretation, the emergent societies of the islands of Fogo and Santiago would have been divided into two major subgroups with very different reproductive success: one composed by the offspring of mixed unions between European men and “domestic” slave women, which later became the major ruling segment of the Cape Verde society; and the other composed by the “rural slaves” who, due to their higher mortalities (both pre- and post-reproductive,) had to be continuously replaced by other enslaved Africans from the mainland. Historical work has shown that the slave labor system was more extreme and lasted longer in Fogo than in Santiago [14]. In addition, it is likely that the relative proportions of “rural slaves” and admixed rulers were higher in Santiago than in Fogo, because of the larger size of the former. In this setting, the higher levels of African ancestry presently observed in Santiago were likely to be caused by demographic and social conditions favoring the attenuation of cultural mediated forms of differential reproductive success [54], [55] between admixed rulers and former slaves.

### Impact of Admixture on Skin Color Variation

To test for the impact of the admixture process on phenotypic variation, we obtained quantitative measurements of skin color, a phenotype that is highly divergent between Cape Verde’s parental populations. There are well-established correlations between skin color and individual ancestry that depend on the admixture dynamics [56], [8], [11] which can also be observed in Cape Verde.

The differences in the admixture proportions among the islands of the archipelago are clearly evident in the skin pigmentation distribution across the islands, since individuals from islands with higher levels of European ancestry tend to have significantly lighter skin colors than individuals from islands where African ancestry predominates (Figure 2). Overall, the correlation between skin color and African ancestry is high, indicating that the distribution of individual African ancestry in Cape Verde is tagging efficiently skin color allele variants, and that this population provides a good model for studying the genetic architecture of pigmentary traits. Mapping studies of pigmentation should include population samples from all the islands in order to fully analyze the whole spectrum of phenotypic and of individual admixture variation in the archipelago. However, there is the trade-off of increased population stratification due to the differences in the admixture levels among islands, which can give rise to spurious associations between the genotypes and the phenotype. This implies that the mapping design has to include a larger numbers of AIMs in order to sufficiently adjust for the admixture stratification.

### Relationship between Skin Color, Genetic Ancestry and SES

We investigated the social impact of skin color in Cape Verde by analyzing its relationship with SES and genetic ancestry. Our study may be considered preliminary, since we only used three categorical variables to evaluate SES. A more thorough evaluation of these variables and of other unmeasured socioeconomic differences and how these affect skin color variation is in order. Notwithstanding, the fact that the correlations analyzed were consistent across all three SES measures and that the results were similar when comparing the different islands strengthens our conclusions. We observed significant correlations between SES and skin color, as measured by reflectometry, after adjusting for genetic ancestry, but no correlations between SES and genetic ancestry. This finding suggests that although genetic ancestry is significantly correlated with skin color, it does not fully capture the effect of skin color on the social dimensions of the contemporary population of Cape Verde.

The perception of skin color as a basis for social stratification was also observed in other admixed populations [26]–[28]. These complex social interactions not only impact the cultural, social, and genetic variation dynamics, but may also have implications for the distribution of genetic and environmental disease risk factors. In one well-documented example, social classification of color has been shown to differ from skin color, as measured by reflectometry, and increased blood pressure was found to be associated with the former, but not with the latter, through interaction with SES [26], [57]. In other instances, reported associations of disease risk with genetic ancestry did not persist after taking socioeconomic variables into consideration, suggesting that ethnic health disparities can be better explained by sociocultural rather than genetic factors [58], [59].

Our observation, together with these results, indicate that culturally perceived color, objective measures of skin pigmentation and genetic ancestry may not always be adequate proxies of each other, and their relationship with socioeconomic risk factors needs to be carefully evaluated to completely understand how human biological diversity shapes variation in disease patterns.

### Genetic Differentiation within the Archipelago

To investigate the genetic relationship among the Cape Verde Islands, we focused on the patterns of NRY variation, since the higher sensitivity of Y chromosome to genetic drift provides adequate resolution to study microevolutionary events occurring since colonization. Moreover, as the maternal contribution was almost exclusively derived from Africa [23], the NRY is more likely to better capture the diverse origins of Cape Verde settlers than mtDNA.

We found that patterns of NRY-UEP variation essentially reflect differences in admixture proportions between islands, while NRY-STR variation reveals additional patterns of population differentiation (Figure 4). One major aspect of this differentiation is the separation of the northern islands of Santo Antão, São Vicente and São Nicolau from the southernmost islands of Santiago and Fogo (Figure 1 and 4). Moreover, whereas the three northern islands likely experienced high levels of gene flow and are closely related to each other, the southern islands Santiago and Fogo are clearly differentiated, in spite of the geographic proximity of the two islands and the presence of founder lineages in Fogo that can be traced to Santiago (Table 3).

Taking into consideration the historical data [14], these patterns could be interpreted in two ways. According to one hypothesis, a substantial part of the North-to-South genetic differentiation can be attributed to demographic events (admixture, drift and founder effects) ensuing the initial settlement of Santiago and Fogo, without further significant exogenous contributions besides the regular importation of slaves to the two southern islands. Alternatively, the North and South genetic clusters could result from separate migrations coming from Europe (mainly Portugal) and the West Coast of Africa, which then evolved in parallel before converging into a common cultural and social background.

Consistent with the first hypothesis, we found that Santiago is the most genetically diverse island of the archipelago (Figure 5), and played particularly important role in the settling of Fogo (Table 3), where evidence for founder effects is especially striking (Figure 5). However, the two peopling hypotheses are not mutually exclusive and we also find a significant genetic component that is exclusive to each island.

All these results, together with the admixture analysis, are concordant in indicating that the most likely scenario for the colonization of the Cape Verde archipelago may lie in between the two stated hypotheses, suggesting that the various groups of islands have a shared genetic history that results from a common origin in Santiago, followed by differentiation through genetic drift and subsequent input of independent external migrations.

In conclusion, our work demonstrates that Cape Verde is a highly admixed population with substantial geographic heterogeneity resulting from different historical and demographic events that have taken place during and since the colonization period.

The wide distribution of individual African ancestry anticipates that Cape Verde holds great potential for analyzing the genetic basis of complex phenotypic traits differing between Africans and Europeans, provided that the study has enough resolution to extract ancestry information to control for population stratification and that differences in SES are carefully taken into account.

## Supporting Information

Figure S1 Patterns of NRY-STR haplotype sharing between the Cape Verde islands. Only Y-chromosomes found to be shared between at least one pair of islands were included in the calculations.
(PDF)

Table S1 Characteristics of the 50 autosomal AIMS. The table shows the physical and genetic locations, frequencies of the reference sequence allele and allele frequency differences between European and West African parental populations (δ).
(DOC)

Table S2 Characteristics of the 34 X-chromosome AIMS. The table shows the physical and genetic locations, frequencies of the reference sequence allele and allele frequency differences between European and West African parental populations (δ).
(DOC)

Table S3 Populations used for Y-chromosome admixture estimation.
(DOC)

Note S1 Assessment of household amenities.
(PDF)
