# Supplementary figures and images for: The Admixture Structure and Genetic Variation of the Archipelago of Cape Verde and Its Implications for Admixture Mapping Studies
Source: PLoS One. 2012 Nov 30;7(11):e51103. doi: 10.1371/journal.pone.0051103 (PMC3511383; doi:10.1371/journal.pone.0051103)

**Figure S1**

**Boa Vista**

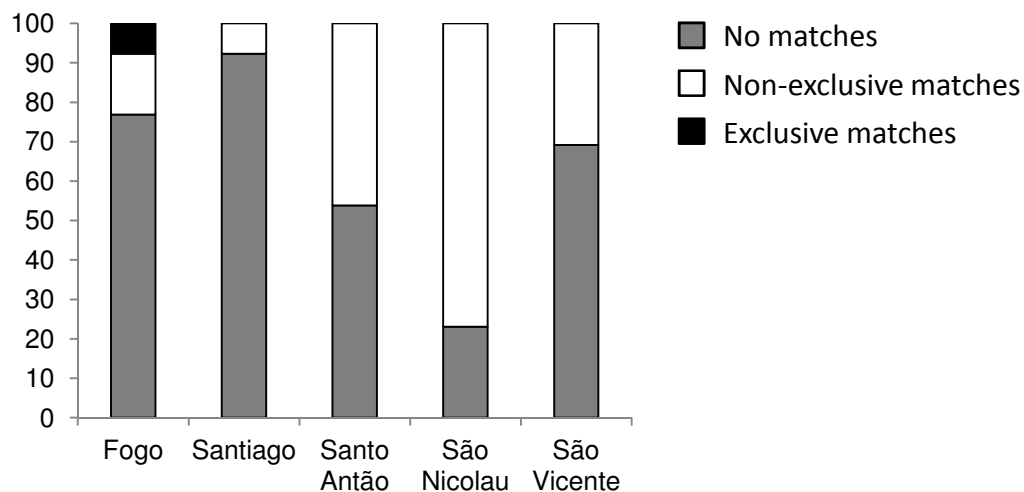

**Santo Antão**

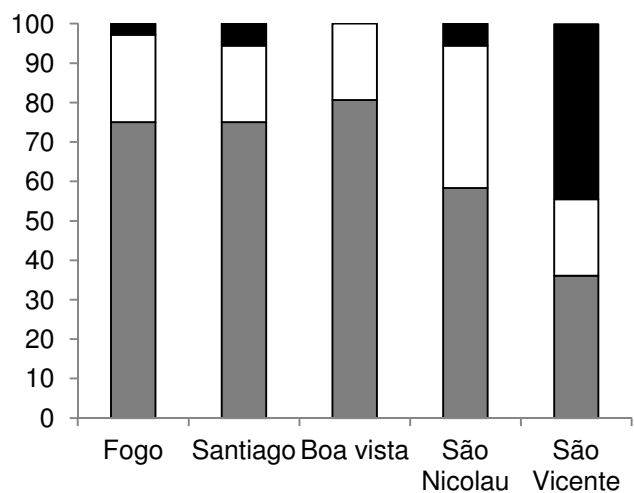

**São Vicente**

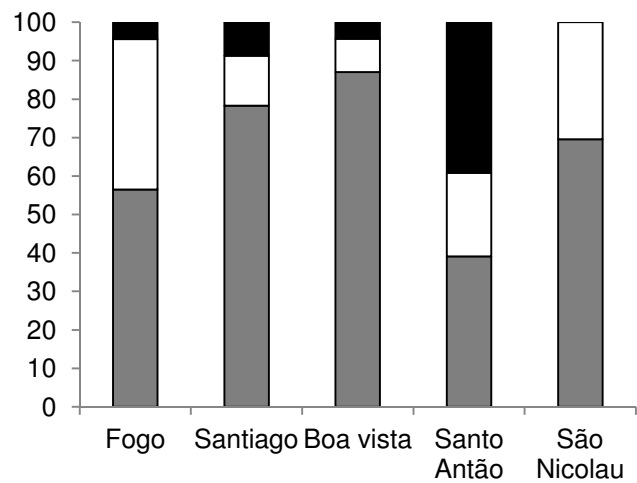

## São Nicolau

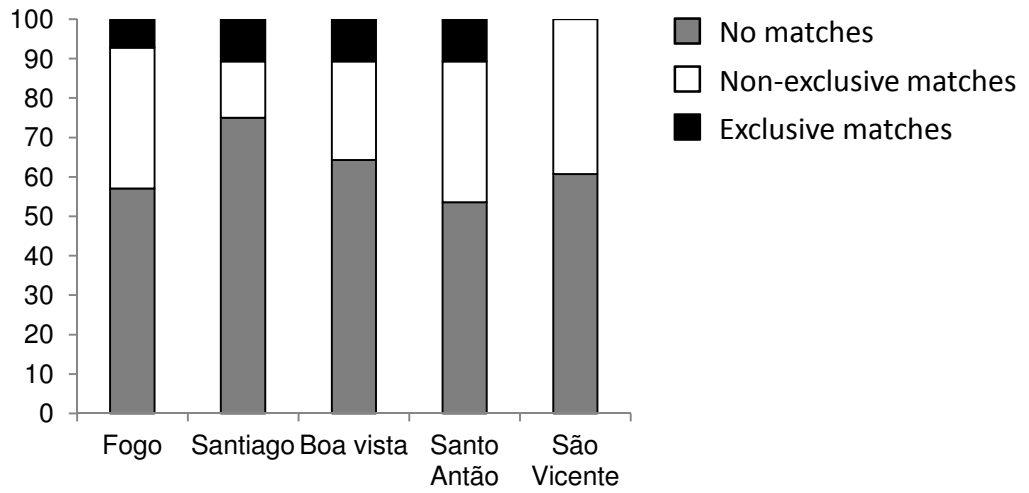

## Fogo

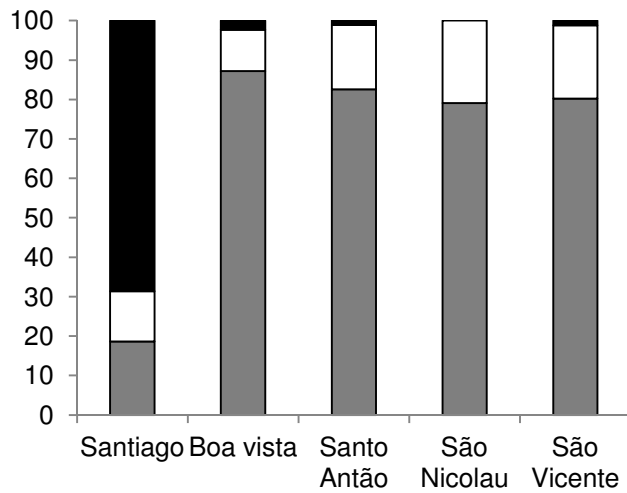

## Santiago

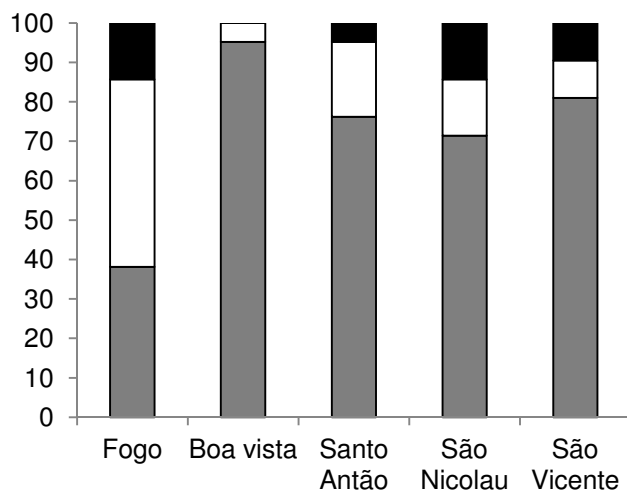

Supplement: Figure S1 — Patterns of NRY-STR haplotype sharing between the Cape Verde islands. Only Y-chromosomes found to be shared between at least one pair of islands were included in the calculations. (PDF) [file pone.0051103.s001.pdf]
