# Supplementary material for: The Admixture Structure and Genetic Variation of the Archipelago of Cape Verde and Its Implications for Admixture Mapping Studies
Source: PLoS One. 2012 Nov 30;7(11):e51103. doi: 10.1371/journal.pone.0051103 (PMC3511383; doi:10.1371/journal.pone.0051103)
Supplement: Table S1 — Characteristics of the 50 autosomal AIMS. The table shows the physical and genetic locations, frequencies of the reference sequence allele and allele frequency differences between European and West African parental populations (δ). (DOC) [file pone.0051103.s002.doc]

**Table S1.** Characteristics of the 50 autosomal AIMS. The table shows the physical and genetic locations, frequencies of the reference sequence allele and allele frequency differences between European and West African parental populations (δ).

| **AIM** | **Chrom**  **location** | **Physical**  **Pos (bp)** | **Genetic**  **Pos (cM)** | **WAfrican** | **European** | **δ** |
| --- | --- | --- | --- | --- | --- | --- |
| rs2225251 | 1p34.2 | 43,301,790 | 71.959 | 0.958 | 0.415 | 0.543 |
| rs725667 | 1p31.3 | 68,261,238 | 99.281 | 0.258 | 0.933 | 0.675 |
| rs7540269 | 1q21.3 | 151,102,633 | 163.348 | 0.465 | 0.992 | 0.527 |
| rs2814778^a^ | 1q23.2 | 157,441,307 | 170.839 | 0.001 | 0.991 | 0.990 |
| rs2340727 | 1q23.3 | 160,213,351 | 174.705 | 0.051 | 0.828 | 0.777 |
| rs725416 | 1q25.2 | 175,414,782 | 193.303 | 0.217 | 0.825 | 0.608 |
| rs6003 | 1q31.3 | 195,297,644 | 208.858 | 0.730 | 0.078 | 0.652 |
| rs832173 | 1q32.1 | 199,524,807 | 212.574 | 0.999 | 0.009 | 0.990 |
| rs1506069 | 1q42.12 | 223,269,819 | 243.293 | 0.075 | 0.992 | 0.917 |
| rs6587361 | 1q42.13 | 228,085,671 | 247.053 | 0.085 | 1.000 | 0.915 |
| rs2776937 | 10p11.22 | 33,646,195 | 58.325 | 0.917 | 0.258 | 0.659 |
| rs2207782 | 10q23.1 | 83,747,179 | 109.145 | 0.125 | 0.825 | 0.700 |
| rs4764216 | 12p12.3 | 15,682,634 | 32.752 | 0.800 | 0.033 | 0.767 |
| rs726391 | 12p12.3 | 18,917,349 | 36.104 | 0.925 | 0.192 | 0.733 |
| rs3782181 | 12q22 | 87,477,692 | 102.034 | 0.800 | 0.186 | 0.614 |
| rs642742^d^ | 12q22 | 87,823,877 | 102.145 | 0.068 | 0.836 | 0.768 |
| rs717091 | 13q14.11 | 43,583,946 | 44.83 | 0.792 | 0.168 | 0.624 |
| rs2078588 | 13q21.33 | 71,199,633 | 66.45 | 0.117 | 0.958 | 0.841 |
| rs1800404^e^ | 15q13.1 | 25,909,368 | 29.189 | 0.883 | 0.265 | 0.618 |
| rs724729 | 15q14 | 37,184,053 | 50.834 | 0.138 | 0.858 | 0.720 |
| rs275183 | 15q14 | 37,232,258 | 50.885 | 0.200 | 0.908 | 0.708 |
| rs1153849 | 15q21.1 | 43,482,987 | 55.236 | 0.094 | 0.719 | 0.625 |
| rs1426654^b^ | 15q21.1 | 46,213,776 | 58.31 | 0.025 | 1.000 | 0.975 |
| rs734780 | 15q26.1 | 87,365,962 | 104.204 | 0.291 | 0.930 | 0.639 |
| rs1003645 | 17q12 | 31,364,397 | 56.713 | 0.966 | 0.192 | 0.774 |
| rs1074075 | 17q22 | 48,608,764 | 74.485 | 0.158 | 0.817 | 0.659 |
| rs3760281 | 17q22 | 51,157,210 | 77.095 | 0.067 | 0.871 | 0.804 |
| rs345162 | 17q23.2 | 55,604,241 | 83.5 | 0.068 | 0.875 | 0.807 |
| rs7211872 | 17q23.2 | 55,905,507 | 83.517 | 0.169 | 0.992 | 0.823 |
| rs1369290 | 18q22.2 | 65,842,500 | 95.953 | 0.925 | 0.033 | 0.892 |
| rs1861498 | 2p25.1 | 7,993,072 | 16.707 | 0.875 | 0.158 | 0.717 |
| rs1526028 | 2p22.1 | 39,635,635 | 61.367 | 0.725 | 0.008 | 0.717 |
| rs10497191 | 2q24.1 | 158,375,463 | 169.412 | 0.975 | 0.100 | 0.875 |
| rs6086279 | 20p12.3 | 7,838,226 | 23.314 | 0.000 | 0.695 | 0.695 |
| rs1465648 | 3p11.2 | 88,032,919 | 108.511 | 0.103 | 0.775 | 0.672 |
| rs17203 | 3p12.3 | 33,987,450 | 103.397 | 0.183 | 0.849 | 0.666 |
| rs16891982^c^ | 5p13.3 | 178,559,215 | 51.779 | 1.000 | 0.049 | 0.951 |
| rs6869589 | 5q35.3 | 178,559,215 | 199.197 | 0.017 | 1.000 | 0.983 |
| rs469318 | 5q35.3 | 179,272,612 | 200.849 | 0.033 | 0.793 | 0.760 |
| rs2077681 | 6p25.2 | 3,031,099 | 7.918 | 0.164 | 0.955 | 0.791 |
| rs222541 | 6q16.1 | 95,287,884 | 101.22 | 0.042 | 0.700 | 0.658 |
| rs1044498 | 6q23.2 | 132,214,061 | 133.612 | 0.000 | 0.873 | 0.873 |
| rs3123101 | 6q25.3 | 159,105,551 | 169.502 | 0.992 | 0.208 | 0.784 |
| rs2396676 | 7q31.1 | 113,114,402 | 127.595 | 0.102 | 0.784 | 0.682 |
| rs7786541 | 7q31.33 | 126,569559 | 134.785 | 0.000 | 1.000 | 1.000 |
| rs2341823 | 7q32.3 | 131,744,403 | 141.353 | 0.108 | 0.925 | 0.817 |
| rs344461 | 7q35 | 146,053,343 | 158.882 | 0.000 | 0.963 | 0.963 |
| rs3176921 | 8q13.1 | 67,253,933 | 83.093 | 0.367 | 0.933 | 0.566 |
| rs2733831^f^ | 9p23 | 12,693,484 | 26.488 | 0.965 | 0.415 | 0.550 |
| rs2789823 | 9q34.2 | 135,759,709 | 172.926 | 0.933 | 0.000 | 0.933 |

Note: Marked AIMs fall within know genes: ^a^*FY-null*; *^b^SLC24A5*; *^c^SLC45A2*; *^d^KITLG*; *^e^OCA2*; *^f^TYRP1*. *FY-null* is the Duffy null blood group allele and *SLC24A5*, *SLC45A2*, *KITLG*, *OCA2*, *TYRP1* are candidate pigmentation genes .

**References:**

1. Parra EJ (2007) Human pigmentation variation: evolution, genetic basis, and implications for public health. Am J Phys Anthropol Suppl 45: 85-105.

2. Tournamille C, Colin Y, Cartron JP, Le Van Kim C (1995) Disruption of a GATA motif in the Duffy gene promoter abolishes erythroid gene expression in Duffy-negative individuals. Nat Genet 10: 224-228.
