# Supplementary material for: The Admixture Structure and Genetic Variation of the Archipelago of Cape Verde and Its Implications for Admixture Mapping Studies
Source: PLoS One. 2012 Nov 30;7(11):e51103. doi: 10.1371/journal.pone.0051103 (PMC3511383; doi:10.1371/journal.pone.0051103)
Supplement: Table S2 — Characteristics of the 34 X-chromosome AIMS. The table shows the physical and genetic locations, frequencies of the reference sequence allele and allele frequency differences between European and West African parental populations (δ). (DOC) [file pone.0051103.s003.doc]

**Table S2.** Characteristics of the 34 X-chromosome AIMS. The table shows the physical and genetic locations, frequencies of the reference sequence allele and allele frequency differences between European and West African parental populations (δ).

| **AIM** | **Physical Pos (bp)** | **Genetic Pos (cM)** | **WAfrican** | **European** | **δ** |
| --- | --- | --- | --- | --- | --- |
| rs211644 | 2,872,721 | 16.0972 | 0.920 | 0.285 | 0.635 |
| rs723259 | 9,799,198 | 25.9111 | 0.720 | 0.041 | 0.679 |
| rs1474458 | 10,957,061 | 27.4971 | 0.264 | 0.890 | 0.626 |
| rs3747295 | 17,656,165 | 28.9016 | 0.067 | 0.943 | 0.876 |
| rs6633170 | 18,847,131 | 54.9589 | 0.051 | 0.779 | 0.728 |
| rs5955757 | 19,283,760 | 58.7220 | 0.035 | 0.980 | 0.945 |
| rs1011759 | 35,242,142 | 70.0445 | 0.919 | 0.269 | 0.650 |
| rs5963039 | 38,234,863 | 70.2236 | 0.202 | 0.897 | 0.695 |
| rs7887756 | 44,668,666 | 74.7468 | 0.295 | 0.923 | 0.628 |
| rs5952682 | 44,851,739 | 78.1626 | 0.312 | 0.923 | 0.611 |
| rs6609827 | 48,818,318 | 78.8266 | 0.341 | 1.000 | 0.659 |
| rs7061449 | 54,036,020 | 79.1381 | 0.129 | 0.994 | 0.865 |
| rs7886355 | 62,875,973 | 79.4273 | 0.056 | 0.962 | 0.906 |
| rs699863 | 65,369,520 | 85.4887 | 0.016 | 0.814 | 0.798 |
| rs492933 | 67,180,569 | 85.5037 | 0.010 | 0.627 | 0.617 |
| rs2209549 | 73,875,354 | 85.6631 | 0.860 | 0.141 | 0.719 |
| rs4892539 | 74,215,399 | 89.2416 | 0.233 | 0.958 | 0.725 |
| rs234256 | 75,870,206 | 100.1831 | 0.249 | 0.988 | 0.739 |
| rs1015736 | 85,575,340 | 104.1408 | 0.204 | 0.957 | 0.753 |
| rs764581 | 95,327,205 | 110.2751 | 0.975 | 0.308 | 0.667 |
| rs1020903 | 99,040,050 | 110.6170 | 1.000 | 0.097 | 0.903 |
| rs768312 | 109,558,040 | 116.3980 | 0.306 | 0.994 | 0.688 |
| rs992864 | 110,387,239 | 120.3416 | 0.067 | 0.943 | 0.876 |
| rs5988008 | 114,864,565 | 136.4160 | 0.152 | 0.818 | 0.666 |
| rs714749 | 117,164,560 | 138.3104 | 0.728 | 0.062 | 0.666 |
| rs766751 | 126,496,367 | 140.6890 | 1.000 | 0.378 | 0.622 |
| rs909657 | 128,611,248 | 147.1259 | 0.143 | 0.912 | 0.769 |
| rs1908816 | 132,808,721 | 170.0899 | 0.978 | 0.16 | 0.818 |
| rs12687833 | 138,669,673 | 170.3605 | 0.261 | 0.936 | 0.675 |
| rs764536 | 147,767,215 | 170.8085 | 0.966 | 0.078 | 0.888 |
| rs758439 | 147,872,587 | 180.4015 | 0.966 | 0.287 | 0.679 |
| rs732604 | 148,190,759 | 180.9831 | 0.275 | 0.938 | 0.663 |
| rs5201 | 152,825,187 | 16.09718 | 0.050 | 0.734 | 0.684 |
| rs12557310 | 154,388,892 | 25.91105 | 0.955 | 0.269 | 0.686 |
