# Supplementary material for: The Admixture Structure and Genetic Variation of the Archipelago of Cape Verde and Its Implications for Admixture Mapping Studies
Source: PLoS One. 2012 Nov 30;7(11):e51103. doi: 10.1371/journal.pone.0051103 (PMC3511383; doi:10.1371/journal.pone.0051103)
Supplement: Table S3 — Populations used for Y-chromosome admixture estimation. (DOC) [file pone.0051103.s004.doc]

**Table S3**. Populations used for Y-chromosome admixture estimation.

| **Geographic area; Pop name** | **Place of origin** | **Linguistic Affiliation** | **n** | **Reference** |
| --- | --- | --- | --- | --- |
| ***European*** |  |  |  |  |
| Portuguese | Portugal | Portuguese | 658 |  |
| Portuguese | Portugal | Portuguese | 138 |  |
| Spanish | Spain | Spanish | 1002 | [2] |
| Sephardic Jews | Portugal; Bulgaria; Djerba;  Greece; Spain; Turkey | Various | 174 | [2] |
|  |  |  |  |  |
| ***North Africa*** |  |  |  |  |
| Morocco | Morocco | Afro-Asiatic | 147 |  |
| Algeria | Algeria | Afro-Asiatic | 46 |  |
| Tunisia | Tunisia | Afro-Asiatic | 139 | [4] |
| Saharawi | Western Sahara | Afro-Asiatic | 29 | [2,3] |
|  |  |  |  |  |
| ***West Africa*** |  |  |  |  |
| Mali (various) | Mali | -^1^ | 44 |  |
| Senegal (various) | Senegal | Niger-Congo | 140 |  |
| Fon | Benin | Niger-Congo | 100 |  |
| Rimaibe | Burkina Faso | Niger Congo | 37 |  |
| Fulbe | Burkina Faso | Niger-Congo | 20 | [8] |
| Mossi | Burkina Faso | Niger-Congo | 49 | [8] |
| Guinea Bissau (various) | Guinea Bissau | Niger-Congo | 282 |  |

^1^Linguistic Affiliation was not mentioned in the reference.

**References:**

1. Beleza S, Gusmao L, Lopes A, Alves C, Gomes I, et al. (2006) Micro-phylogeographic and demographic history of Portuguese male lineages. Ann Hum Genet 70: 181-194.

2. Adams SM, Bosch E, Balaresque PL, Ballereau SJ, Lee AC, et al. (2008) The genetic legacy of religious diversity and intolerance: paternal lineages of Christians, Jews, and Muslims in the Iberian Peninsula. Am J Hum Genet 83: 725-736.

3. Bosch E, Calafell F, Comas D, Oefner PJ, Underhill PA, et al. (2001) High-resolution analysis of human Y-chromosome variation shows a sharp discontinuity and limited gene flow between northwestern Africa and the Iberian Peninsula. Am J Hum Genet 68: 1019-1029.

4. Arredi B, Poloni ES, Paracchini S, Zerjal T, Fathallah DM, et al. (2004) A predominantly neolithic origin for Y-chromosomal DNA variation in North Africa. Am J Hum Genet 75: 338-345.

5. Underhill PA, Shen P, Lin AA, Jin L, Passarino G, et al. (2000) Y chromosome sequence variation and the history of human populations. Nat Genet 26: 358-361.

6. Semino O, Santachiara-Benerecetti AS, Falaschi F, Cavalli-Sforza LL, Underhill PA (2002) Ethiopians and Khoisan share the deepest clades of the human Y-chromosome phylogeny. Am J Hum Genet 70: 265-268.

7. Luis JR, Rowold DJ, Regueiro M, Caeiro B, Cinnioglu C, et al. (2004) The Levant versus the Horn of Africa: evidence for bidirectional corridors of human migrations. Am J Hum Genet 74: 532-544.

8. Cruciani F, Santolamazza P, Shen P, Macaulay V, Moral P, et al. (2002) A back migration from Asia to sub-Saharan Africa is supported by high-resolution analysis of human Y-chromosome haplotypes. Am J Hum Genet 70: 1197-1214.

9. Rosa A, Ornelas C, Jobling MA, Brehm A, Villems R (2007) Y-chromosomal diversity in the population of Guinea-Bissau: a multiethnic perspective. BMC Evol Biol 7: 124.
