# Supplementary material for: The Admixture Structure and Genetic Variation of the Archipelago of Cape Verde and Its Implications for Admixture Mapping Studies
Source: PLoS One. 2012 Nov 30;7(11):e51103. doi: 10.1371/journal.pone.0051103 (PMC3511383; doi:10.1371/journal.pone.0051103)
Supplement: Note S1 — Assessment of household amenities. (PDF) [file pone.0051103.s005.pdf]

Supplementary note: Assessment of household amenities

The SES is a multidimensional construct, comprising elements related with wealth and prestige in the society. Various variables have been used to reflect this concept, but these variables have different meanings across populations with different economic, social and cultural structures [1].

In Cape Verde, there are marked contrasts in the dwelling features among the social strata. For this reason, the census of the country's National Institute of Statistics includes a section of 17 multiple-choice questions to assess the quality of household amenities of the citizens' home (Table SN1). We have reproduced those questions in our demographic questionnaires.

Table SN1. List of multi-choice questions to assess household amenities included in the Cape Verdean Census and reproduced in our demographic questionnaires.

**Questions to assess quality of household amenities**
1. Dwelling type.
2. What is the house's wall material?
3. What is the house's roofing material?
4. What is the house's floor material?
5. How many rooms are in the house?
6. Does the house have a kitchen?
7. What is the main source of water supply in your house?
8. How long does it take to get to the water source?
9. Which means of transportation do you use for get to the water supply?
10. Features of bathroom (presence or not of bathroom, presence or not of toilet, type of toilet).
11. How is the discharge of the residual waters?
12. What is the main source of energy for illumination?
13. What is the main source of energy for food preparation?
14. The house that you live in is rented or owned?
15. Which essential equipment belongings (stove, fridge…) do you have?
16. Which other equipment belongings (TV, radio, computer, …) do you have?
17.Which means of transportation does your family aggregate possess?

Of the list of questions in table SN1, we discarded from the analysis those for which more than 90% of the volunteers answered in the same way (questions 1,14 and 15 in table SN1) and for which there was a substantial amount of missing data (questions 5, 8, and 9 on table SN1, where, for several reasons, volunteers had difficulties in answering). At the end, we considered 10 questions for analyses. We scored the answers of each question from 1 to the total number of choices. Due to significant correlations among these 10 household amenity variables, we carried out principal component analysis (PCA) to obtain summarized measures of SES that were not collinear. We then used the scores on the two first principal components as independent variables in multiple regression models on the M index and on the estimated proportion of West African ancestry. The analyses were performed in the R statistical computing environment.

References:

1. Braveman P, Cubbin C, Marchi K, Egerter S, Chavez G (2001) Measuring socioeconomic status/position in studies of racial/ethnic disparities: maternal and infant health. Public Health Rep 116: 449–463.
